# SARS-CoV-2: vaccines in the pandemic era

**DOI:** 10.1186/s40779-020-00296-y

**Published:** 2021-01-06

**Authors:** Dan-Dan Li, Qi-Han Li

**Affiliations:** Institute of Medical Biology, Chinese Academy of Medicine Science & Peking Union Medical College, Yunnan Key Laboratory of Vaccine Research and Development on Severe Infectious Diseases, No. 935 Jiaoling Road, Kunming, 650118 Yunnan China

**Keywords:** SARS-CoV-2, COVID-19, Vaccine, Vaccine candidate, Clinical trials

## Abstract

Coronavirus disease 2019 (COVID-19), caused by the severe acute respiratory syndrome coronavirus 2 (SARS-CoV-2), has caused millions of infections and deaths worldwide since its emergence in December 2019. As there is little or no natural immunity in the human population or specific anti-COVID-19 drugs, researchers from the government, academia and industry are developing vaccines at an unprecedented speed to halt the pandemic. In this review, the results of animal experiments and clinical trials on several vaccine technical platforms are summarized, and several challenges are also discussed to further promote the development, evaluation and application of vaccines during the challenging situation of the global pandemic.

## Background

In December 2019, an outbreak of acute pneumonia with unknown etiology was first reported in Wuhan, China. The pathogen, which causes a disease known as coronavirus disease 2019 (COVID-19), was identified as a new coronavirus and was subsequently named severe acute respiratory syndrome coronavirus 2(SARS-CoV-2). The World Health Organization declared the outbreak a Public Health Emergency of International Concern on January 30, 2020 and declared it a pandemic on March 11 [1, 2]. As of December14, 2020, more than 70 million cases of COVID-19 have been reported in more than 188 countries and territories [3], resulting in more than 1 million deaths, along with global social and economic disruption. With the primary treatments remaining symptomatic and supportive, an effective and adequate vaccine is the ultimate strategy for humans to overcome this pandemic.

**Fig. 1:**
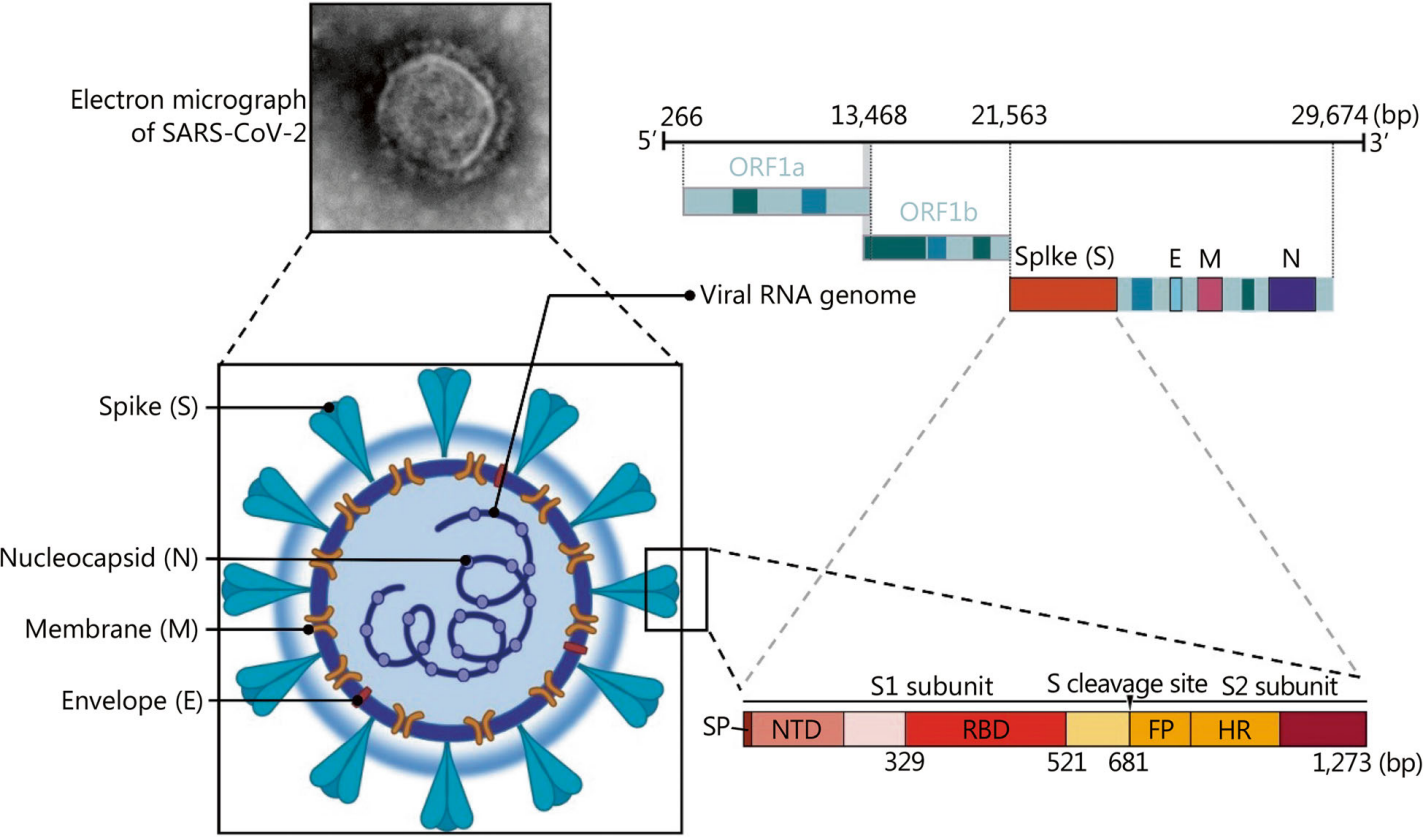
The basics of SARS-CoV-2 and an in-depth look into the SARS-CoV-2 spike glycoprotein. Electron micrograph showing the whole SARS-CoV-2 virion. Four main structural proteins, S, M, N, and E, are labelled; details of the RNA genome and spike gene are shown. S. Spike; N. Nucleocapsid; M. Membrane; E. Envelope; ORF. Open reading frame; SP. Signal peptide; NTD. N-terminal domain; RBD. Receptorbinding domain; FP. Fusion peptide; HR. Heptad repeats

**Fig. 2:**
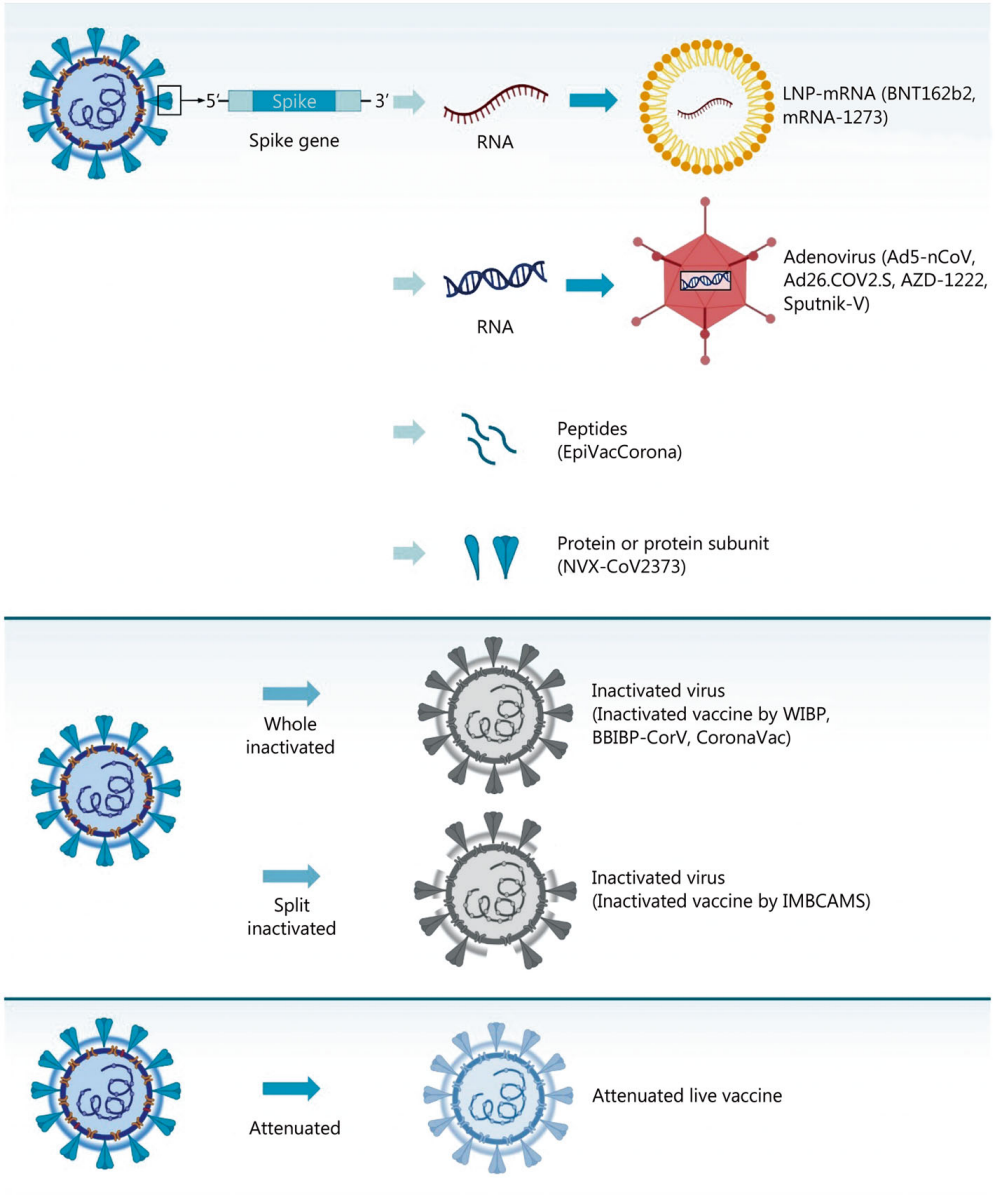
Various platforms of vaccine candidates for COVID-19. mRNA, adenovirus vector, peptide and protein subunit vaccines have been developed based on the genetic information of SARS-CoV-2, while inactivated vaccines have been developed based on the inactivation of the live wild-type SARS-CoV-2 virus. Attenuated live vaccines are developed based on attenuation of the wild-type virus. WIBP. Wuhan Institute of Biological Products; IMBCAMS. Institute of Medical Biology, Chinese Academy of Medicine Sciences

SARS-CoV-2 belongs to the broad family of viruses known as coronaviruses and is a member of the subgenus Sarbecovirus (betacoronavirus lineage B) [4]. Seven members of the virus family are known to have the ability to infect humans, and three of them cause severe respiratory diseases, including SARS virus (now known as SARS-CoV-1) and Middle East respiratory syndrome coronavirus (MERS-CoV) [5]. SARS-CoV-2 has a genome of 29.8–29.9 kb [6, 7]. The SARS-CoV-2 virion is generally spherical with a diameter of 60–140 nm and has a unique spike length of 9–12 nm on the virus particle surface, which is composed of four structural and nonstructural proteins (NSPs) (Fig.1) [8]. The structural proteins of coronaviruses play an important role in viral assembly and host infection. Trimers of the S protein are highly glycosylated and form spikes on the surface of the viruses that are responsible for binding to host cell receptors and allowing the coronavirus to invade host cells [9]. The M protein has three transmembrane domains, causing it to bend and assume a spherical shape, maintaining the basic shape of the virus particle [10]. The E proteins are involved in viral assembly and release [11]. When coronaviruses infect host cells, they bind to host cell receptors through the receptor-binding domain (RBD) in the S1 subunit of the spike protein, and the S2 subunit mediates fusion between the virus and the cell membrane. The genomic homology of SARS-CoV-2with SARS-CoV is as high as 79.5% [12]. Wan et al. [12] and Zhou et al. [13] demonstrated through structural analysis and cell experiments, respectively, that SARS-CoV-2 utilizes ACE2 as a cell receptor like SARS-CoV. Since neutralizing antibodies against the S protein block virus entry into host cells [14], most COVID-19 vaccine candidates have been designed using the S protein as the primary antigen (Fig. 2). We have summarized a series of representative vaccine candidates and their primary characteristics in Table 1.

**Table 1:** Representative vaccine candidates and their primary characteristics

Vaccine name	Developer	Type	Antigens	Preclinical studies	Clinical studies	Latest updates	No. of subjects (*n*)	Age of subjects (year)	Procedure	Dosage	GMT for NAbs (peak-value)	Human. Conv. Serum	Method for NAbs
**mRNA vaccine**													
BNT162b1	BioNTech/Pfizer	LNP-mRNA	RBD trimer	N.A.	Phase 1/2 [20]:		45	18–55	0/21	10, 30, 100 μg	168, 267, (N.A)	94	VNT_50_
BNT162b2			S-full length (pre-fusion)	Mice, Monkeys [24] (Challenge: Yes)	Phase 1/2 [21]:		60	18–55	0/21	1, 10, 30, 50 μg	36, 158, 308, 578	94	
					Phase 1 (b1&b2) [22, 23]:		105	18–55	0/21	b1: 10, 20, 30, 100 μg b2: 10, 20, 30 μg	b1: 180, 203, 437, (N.A) b2: 157, 363, 361	94	
							90	65–85		b1: 10, 20, 30 μg b2: 10, 20, 30 μg	b1:33, 179, 101 b2:111, 84, 206		
					phase 3: NCT04368728, interim analysis of BNT162b2 released and showed 90% effectiveness [26].								
mRNA-1273	Moderna	LNP-mRNA	S-full length (pre-fusion)	Mice [22] Monkeys [31] (Challenge: Yes)	Phase 1 [27]:		45	18–55	0/28	25, 100, 250 μg	112, 343, 373	109	PRNT
					phase 1 [28]:		20	56–70	0/28	25, 100 μg	116, 402	106	
							20	> 71		25, 100 μg	121, 317		
					phase 3: NCT04470427								
**Viral vector vaccine**													
Ad5-nCoV	CanSino	Ad5	S-full length	Mice [38] (Challenge: Yes)	Phase 1 [36]:		108	18–60	1 dose	5 × 10^10^, 1 × 10^11^, 1.5 × 10^11^ vp	14.5, 16.2, 34	N.A.	live virus neutralisation, pseudovirus neutralisation tests
					Phase 2 [37]:		508	18–60	1 dose	5 × 10^10^, 1 × 10^11^ vp	18.3, 19.5		
					Phase 3: NCT04526990, NCT04540419								
Ad26.COV2.S	Janssen	Ad26	S-full length (pre-fusion)	Mice [41] Monkeys [40] (Challenge: Yes)	Phase 1/2a [42]:		402	18–55	0/56	5 × 10^10^ vp; 1 × 10^11^ vp	214, 243	522	wild-type virus neutralization assay
							394	> 65	0/56	5 × 10^10^ vp; 1 × 10^11^ vp	196, 127		
					Phase 3: NCT04505722								
AZD-1222	Oxford/AstraZeneca	ChAdOx1-S	S-full length	Mice [44, 45] Monkeys [44] (Challenge: Yes)	Phase 1/2 [46]:		543	18–55	1 dose	5 × 10^10^ vp	87.9	approx. 450	pseudotyped virus neutralisation
							534	18–55	1 dose	standard dose	40		
							10	18–55	0/28	5 × 10^10^ vp	450.9		
					Phase 3: ISRCTN89951424, NCT04516746, NCT04540393, CTRI/2020/08/027170								
Sputnik-V	Gamaleya	rAd26 + rAd5	S-full length	N.A.	Phase 1 [48]		9	18–60	1 dose	rAd26 (Fro, Lyo): 1 × 10^11^ vp	4.29, 3.67	32.96	Microneutralization assay
							9	18–60	1 dose	rAd5 (Fro, Lyo): 1 × 10^11^ vp	6.3, 10.8		
					Phase 2 [48]		20	18–60	0/21	(rAd26)/ (rAd5): (Fro, Lyo): 1 × 10^11^ vp	49.25, 45.95		
					Phase 3: NCT04530396, NCT04564716								
**Recombinant protein vaccine**													
NVX-CoV2373	Novavax	Protein Subunit	S-full length (pre-fusion)	Mice [50] Monkeys [51] (Challenge: Yes)	Phase 1 [49]:		131	18–60	0/21	5 μg,25 μg	3906, 3305	983	Microneutralization assay
						Phase 3: 2020–004123-16 NCT04611802							
EpiVacCorona	Vector	Peptide	SARS-CoV-2 protein	N.A.	Phase 1: NCT04527575		14	18–60	0/21	No published results			
							86	18–60	0/21				
**Inactivated vaccine**													
Inactivated vaccine	Wuhan Institute of Biological Products	Inactivated	Inactivated SARS-CoV-2 virus	N.A.	Phase 1 [55]:		96	18–59	0/28/56	2.5, 5, 10 μg	316, 206, 297	N.A.	PRNT_50_
					Phase 2 [55]:		224	18–59	0/14	5 μg	121		
									0/21	5 μg	247		
					Phase 3: ChiCTR2000034780, ChiCTR2000039000								
BBIBP-CorV	Beijing Institute of Biological Products	Inactivated	Inactivated SARS-CoV-2 virus	Mice,Monkeys [56] (Challenge: Yes)	Phase 1 [57]:		192	18–59	0/28	2, 4, 8 μg	87.7, 211.2, 228.7	N.A.	Infectious SARS-CoV-2 neutralising assay
								60–80	0/28	2, 4, 8 μg	80.7, 131.5, 180.9		
					Phase 2 [57]:		448	18–59	0/14	4 μg	169.5		
									0/21	4 μg	282.7		
									0/28	4 μg	218		
									1 shot	8 μg	14.7		
					Phase 3: ChiCTR2000034780 NCT04560881								
CoronaVac	Sinovac	Inactivated	Inactivated SARS-CoV-2 virus	Mice, Monkeys [58] (Challenge: Yes)	N.A.	Phase 2 [59]:	600	18–59	0/14	3, 6 μg	27.6, 34.5	N.A.	Cytopathogenic effect assay
									0/28	3, 6 μg	between 32 and 64.		
					Phase 3: NCT04456595, 669/UN6.KEP/EC/2020, NCT04582344								
Inactivated vaccine	Institute of Medical Biology, Chinese Academy of Medical Sciences	Inactivated	Inactivated SARS-CoV-2 virus	Mice, Monkeys [60] (Challenge: Yes)	Phase 1 [33]:		192	18–59	0/14	50, 100, 150 EU	18, 54.5, 37.1	N.A.	Infectious SARS-CoV-2 Cytopathogenic effect assay
									0/28	50, 100, 150 EU	10.6, 15.4, 19.6		
					Phase 1/2: NCT04470609								

## mRNA and DNA vaccines

In the event of a pandemic, mRNA and DNA vaccines represent the most quickly accessible vaccine candidates due to their short-term development and low-level biosafety requirements [15–18]. Before this pandemic, due to the low stability and uncertainty surrounding the formulation of mRNA vaccines, no mRNA vaccine candidates were successfully commercialized [19]. With recent technical progress, a number of institutions worldwide have quickly begun work on mRNA and DNA vaccines.

Pfizer and BioNTech developed an mRNA vaccine called BNT162b1, a lipid-soluble nanoparticle preparation comprised of mRNA encoding the S protein RBD trimer [20]. Results of its phase 1 clinical trial showed that subjects in all three dose groups produced high titers of antibody against the RBD and relatively high and moderate titers of neutralizing antibody in the serum. The vaccine was safe and well tolerated in general, although some patients had mild-to-moderate injection site pain and other mild-to-moderate adverse reactions. This study was also the first published clinical trial based on mRNA vaccine technology. Another study showed that doses of both 1 and 50 μg of vaccine induced a strong CD4^+^ and CD8^+^ T cell response, with Th1 CD4^+^ T cells showing a strong reaction, RBD-specific CD4^+^ and CD8^+^ T cells being significantly amplified, and interferon γ (IFN-γ) being significantly secreted. This study provides important data on the T cell response induced by BNT162b1 [21]. Pfizeralso compared the immune efficacy of BNT162b1 to that of BNT162b2, another vaccine candidate, in subjects from two age groups [22, 23]. The difference between BNT162b1 and BNT162b2 is that the antigen of BNT162b1 is a trimerized RBD, while BNT162b2 contains the prefusion conformation of the full-length S gene. In theory, BNT162b2 should be more immunogenic. The results showed that both BNT162b1 and BNT162b2 dose-dependently induced similar serum neutralizing antibody titers that were significantly higher than those in convalescent sera. However, BNT162b2 produced milder adverse reactions than BNT162b1 and BNT162b2 was also effective in elderly individuals. As a result, Pfizer and BioNTech will develop BNT162b1 and BNT162b2 at the same time and rapidly proceed to phase 2/3 clinical trials of BNT162b2 [23]. The results of animal trials for BNT162b2 showed that in both mice and rhesus monkeys, BNT162b2 produced strong Th1-type CD4^+^ and IFN-γ^+^ CD8^+^ T cell responses and was able to completely protect the lungs of rhesus monkeys from SARS-CoV-2 infection [24]. Phase 3 trials of BNT162b2 are currently underway in the United States, Argentina, Brazil, South Africa and Turkey, and approximately 44,000 people will be recruited [25]. On November 9, Pfizer reported the latest progress of the phase 3 clinical trial of BNT162b2, which is the first published population-protection data for a COVID-19 vaccine [26]. Interim analysis indicated that 94 confirmed cases of COVID-19 had occurredin trial participants and that two doses of BNT162b2 at a 21-day interval reduced the infection rate of symptomatic COVID-19 by 90% compared withplacebo. No serious adverse reactions have been observed so far. As a result, Pfizer announced that it had submitted an Emergency Use Authorization (EUA) to the Food and Drug Administration (FDA) and planned to close the clinical trial after an estimated 164 confirmed cases of COVID-19 in trial participants to further characterize the vaccine candidate’s performance.

Another mRNA platform-based vaccine candidate is mRNA-1273, which was developed by the US-based biotechnology company Moderna. mRNA-1273 is a novel lipid nanoparticle (LNP)-encapsulated mRNA-based vaccine that encodes a full-length, prefusion stabilized S protein. mRNA-1273 is the first vaccine candidate subjected to a phase 3 clinical trial. According to the disclosed phase 1 interim report, there was a strong dose-dependent antibody reaction to the S protein after the first and second inoculations; neutralizing serum was found in all subjects after the second inoculation, and the titers were equal to or greater than the serum neutralization titers of recovered COVID-19 patients [27]. The vaccine was safe in general, but some of the subjects experienced adverse reactions. The safety and immunogenicity data for mRNA-1273 from an elderly population showed that mRNA-1273 exhibited good safety and tolerability [28]. No vaccine-related serious adverse events (SAEs) were observed. Four weeks after the second dose, neutralizing antibody titers were similar in subjects of different ages. Besides, a rapid increase in the anti-S antibody titer occurred after the first immunization. The vaccine also elicited a strong Th1 cell response [29]. Animal studies did not show any evidence of enhanced incidence of immune disease in mice [30] or rhesus macaques [31], while the vaccine protected immunized animals against viral challenge. Currently, a phase 3 clinical trial of mRNA-1273 is underway in the United States. 30,000 subjects will be recruited for the phase 3 clinical trial, and partial interim data will be available in late December this year [32].

.Zhang et al. [33] from the Chinese PLA Academy of Military Medical Sciences developed an mRNA vaccine called ARCoV using the S protein RBD as an antigen. The results of animal studies showed that intramuscular immunization with ARCoV mRNA-LNPs (2 μg or 10 μg) induced RBD-specific IgG neutralizing antibodies in mice, and the titer was significantly increased after boosting immunization. A Th1 cell response was also induced. The vaccine protected mice from SARS-SoV-2 virus challenge. In monkeys, results were similar to those observed in mice, with high titers of neutralizing antibodies and Th1-based cellular responses. In addition, the vaccine is stable enough to be stored at 4 °C or 25 °C for one week without affecting delivery efficiency; after storage at 37 °C for one week, efficiency was reduced by only 13%, providing a great advantage in storage and transport of the vaccine products. ARCoV was approved for a phase 1 clinical trial (ChiCTR2000034112) on June 19, 2020.

Inovio Pharmaceuticals published the results of animal studies for its DNA vaccine candidate INO-4800 [34]. A sequence encoding the S protein was inserted into the pGX9501 vector. The vaccine has good antigenicity and immunogenicity in mice and guinea pigs, while in vitro results showed that the induced antibodies can effectively block viral infection. The results of SARS-CoV-2 challenge experiments in rhesus macaques [35] subsequently showed that the vaccine-induced T cell response and neutralizing antibodies were effective against both D614 and G614 strains. A few months after vaccination, S protein-specific T cells and B cells were rapidly activated in response to virus challenge, and the viral load was significantly reduced, suggesting that the DNA vaccine INO-4800 provides sustained humoral and cellular immunity. Phase 1/2 clinical trials of the vaccine are currently underway (NCT04447781, NCT04336410).

## Viral vector vaccines

Ad5-nCoV, the Ad5 adenovirus vector vaccine developed by CanSino Biological Inc. and Beijing Institute of Biotechnology, is the world’s first vaccine candidate to release clinical trial results [36]. An optimized full-length S protein was used as the immunogen of the vaccine. The vaccine was found to induce both humoral and cellular immunity. Most adverse reactions were mild-to-moderate injection site pain. No severe adverse reactions were observed within 28 days after inoculation. Phase 2 clinical trial resultsshowed that RBD-specific antibody levels in the high-dose and low-dose groups in response to single intramuscular immunization peaked 28 days after immunization and that the seroconversion rates of serum antibodies were as high as 96 and 97% [37]. Within 14 days after immunization, 72% of subjects in the high-dose group and 74% of subjects in the low-dose group reported at least one solicited adverse reaction, including pain at the injection site, fever, headache and fatigue. No severe adverse reactions were observed throughout the 28-day observation period. Theresults of animal experiments showed that Ad5-nCoV fully protected the upper and lower respiratory tracts from SARS-CoV-2 infection and that single intramuscular inoculation protected the lungs of mice from SARS-CoV-2 infection and reduced viral replication in the respiratory tract of mice and ferrets [38]. This study also showed that combination of an intramuscular immunization-mediated rapid systemic immune response and mucosal immunization-mediated local mucosal immunity may be more effective than a single vaccination. Phase 3 clinical trials of Ad5-nCoV are currently underway.

Because of the widespread presence of adenovirus, the presence of preexisting antibodies in some populations may impair the efficacy of adenovirus-based vaccines. The hAd5 vaccine developed by Immunity Bio has the potential to provide protection in patients with preexisting antibodies against adenovirus [39]. Using an optimized S protein and a conserved N protein as the antigens and a new-generation hAd5 vector with deletions of E1, E2b and E3, the immune response of preexisting Ad5 antibodies to the vaccine was reduced.

Janssen Vaccines & Prevention developed an Ad26 adenovirus vector vaccine contains variants of the S protein. The vaccine required only a single dose of immunization. It effectively induced high titers of neutralizing antibodies 2 and 4 weeks after immunization in monkeys and produced complete protection against viral infection [40]. The virus was almost completely undetectable in the lower respiratory tract of all groups, and the titer of neutralizing antibody was significantly related to the level of protection. The induced cell response was Th1-biased rather than Th2-biased, which also demonstrating the safety of the vaccine. Immunogenicity and antigenicity tests in mice revealed that optimization of the S protein (with furin and two proline mutations) increased the proportion of neutralizing antibody binding to nonneutralizing antibody binding [41]. This study confirmed that optimization of the S protein improved the protective effect of the vaccine. According to published preliminary clinical trial results, the vaccine was safer in elderly individuals than in younger individuals. For cellular immunity, more than 80% subjects in elderly and younger individuals were positive for Th1 cytokines producing S-specific CD4^+^ T cell responses, with no or very low Th2 responses [42]. The vaccine is currently in a phase 3 clinical trial (NCT) that is expected to recruit 60,000 subjects [43].

Oxford University and AstraZeneca developed a chimpanzee adenovirus (ChAd) recombinant vaccine, ChAdOx1-nCoV-19 (formally named AZD-1222), which includes a codon-optimized S protein gene inserted into the ChAd replication-defective mutant ChAdOx1 [44]. According to published data from animal studies, the vaccine was so effective that a single dose induced highly effective humoral and cellular immunity. Subsequent inoculation in rhesus macaques showed that AZD-1222 induced a high titer of neutralizing antibody and IFN-γ secretion. After challenging with SARS-CoV-2, the viral load in vaccinated monkeys was significantly lower than in control animals, and pathology results showed that there was no pneumonia in vaccinated rhesus monkeys and that an antibody-dependent enhancement (ADE) effect was not observed. Immunogenicity test of AZD-1222 in aged mice showed that a single inoculation induced both cellular and humoral immunity in aged mice, but the extent was lesser than that in young mice (3 weeks) [45]. Boost inoculation significantly enhanced the immune response to the vaccine in aged mice. Together, these results indicate that the immunogenicity of AZD-1222 can be enhanced in older individuals through the use of a prime-boost vaccination strategy. In the published phase 1/2 results, twenty-eight days after inoculation, local and systemic adverse events, such as fatigue, headache and local pain, occurred in the vaccine group, but most of these effects improved without serious adverse events. The neutralizing antibody response was stronger in the boost vaccination group. The vaccine also elicited a T cell response in all subjects. The boost vaccination group did not have an enhanced cellular response, but the group size was too small to make definitive conclusions [46]. Based on the clinical trial plan released by AstraZeneca, there will be a total of 30,000 people enrolled for phase 3 [47].

The COVID-19 vaccine Sputnik-V, which was developed by the Gamaleya Research Institute of Russia, has attracted worldwide attention due to its controversially accelerated approval. According to the published results of a phase 1/2 study, Sputnik-V is a recombinant Ad26 and Ad5 adenovirus vector combined vaccine, and the full-length S gene was used as an antigen. The safety and immunogenicity of two formulations of the vaccine, frozen (Gam-COVIDVac) and lyophilized (Gam-COVID-Vac-Lyo), were evaluated. The vaccine was generally safe, and the most common adverse reactions were injection site pain and fever. All subjects developed antibodies against S protein. The geometric mean titers (GMT) of neutralizing antibodies was not significantly different from that of convalescent patients with a seroconversion rate of 100%. In addition, all subjects exhibited a cellular response on day 28. The phase 3 trial of Sputnik-V was approved on August 26, and 40,000 subjects are planned to be recruited [48].

## Recombinant protein vaccines

Novavax developed the NVX-CoV2373 vaccine, which is the first recombinant protein vaccine with a published clinical trial. Full-length recombinant trimeric S protein expressed and purified in insect cells was nanoparticle coated and tested in a phase 1/2 clinical trial [49]. There were no significant adverse reactions in the clinical trial, and the antibody and neutralization responses in serum reached a peak 35 days after the first inoculation. The vaccine also induced a Th1 CD4 T cell response, and the amount of S protein-specific IgG induced by the vaccine was positively correlated with serum neutralization. In brief, the vaccine induced a high serum neutralizing antibody titer that exceeded that induced by mRNA vaccines, suggesting that NVX-CoV2373 may be the most effective vaccine developed so far. In mice and baboons, NVX-CoV2373 was mixed with Matrix-M adjuvant to induce high-titer S protein-specific IgG, and the vaccine protected mice from SARS-CoV-2 infection, with no evidence of immune-enhancing disease [50]. The vaccine induced CD4^+^ and CD8^+^ T cell responses and the formation of Th cell germinal centers. Immunogenicity and protection were also tested in cynomolgus monkeys, and the results revealed effective protection of the upper and lower respiratory tracts from viral infection, with no evidence of lung disease [51]. These results support the ongoing phase 1/2 clinical trial for NVX-CoV2373. In addition, structural analysis showed that the S protein of the vaccine had a stable prefusion conformation and unique S1 subunit structure and that the S protein trimers interacted with each other to form a more complicated S protein complex. This study demonstrates that the antigen chosen for NVX-CoV2373 possesses structural integrity and biochemical function and is therefore a good vaccine candidate [52].

EpiVacCorona, developed by FBRI SRC VB VECTOR is Russia’s second COVID-19 vaccine. The vaccine uses a chemically synthesized peptide of the SARS-CoV-2 protein as an antigen conjugated to a carrier protein with an aluminum adjuvant and is administered twice at a 21-day interval. The vaccine has entered phase 1/2 clinical trials [53]. No results have been published on this vaccine, but according to media reports, EpiVacCorona developers said that subjects have developed sufficient protective antibodies to last up to six months. The phase 2 clinical trial for EpiVacCorona was said to be completed in September, with the registration process completed in October, production in November and a subsequent post-registration phase 3 clinical trial [54].

## Inactivated vaccines

Inactivated vaccines are one of the most traditional approaches to vaccine development, and due to their simplicity, they are often used as a priority strategy during acute infectious disease outbreaks.

The Wuhan Institute of Biological Products developed an inactivated vaccine. The published results of a phase 1/2 clinical trial showed that the adverse reactions to the vaccine were mild and that the induction of neutralizing antibodies in serum was highly significant [55]. The vaccine was relatively safe and well tolerated by subjects. No severe adverse reactions occurred. The most common adverse reaction was injection site pain, followed by fever. A phase 3 trial was carried out in the United Arab Emirates and Morocco.

The inactivated BBIBP-CorV vaccine was developed by the Beijing Institute of Biological Products. Published animal studies showed that the vaccine, inactivated with β-propyl lactone, induces high titers of neutralizing antibodies in mice, rats, guinea pigs, rabbits, cynomolgus monkeys and rhesus monkeys [56]. In addition, there was no ADE in the lung tissue of immunized monkeys. Toxicity and histopathology studies alsodemonstrated normal results in rats and guinea pigs, and long-term toxicity in cynomolgus monkeys (36 days) was normal as well. In phase 1 trials,29% in the vaccine group experienced at least one adverse reaction 7 days after inoculation that were primarily mild-to-moderate, and no serious adverse reactions occurred within 28 days of inoculation [57]. The serum antibodies of the vaccine group were all positive after inoculation. In phase 2 clinical trials, one subject inoculated with 4 μg had a grade 3 adverse reaction (fever) but later recovered, while the other subjects had mild-to-moderate adverse reactions. The results showed that the BBIBP-CorV vaccine was safe and well tolerated in all groups, that neutralizing antibodies were produced in all subjects 42 days after prime inoculation, and that the strongest neutralization GMT occurred at a dose of 4 μg with the 0/21 or 0/28 day inoculation schedule. Phase 3 trials of BBIBP-CorV are currently underway in Argentina and the United Arab Emirates.

The inactivated CoronaVac vaccine (formerly called PiCoVacc) developed by Sinovac is a multivalent vaccine that targets SARS-CoV-2 strains circulating in several regions [58]. The viral seed was cultured in Vero cells, inactivated with β-propyl lactone. According to the results in mice and rats, the neutralization titer in serum of vaccination group was significantly higher than that in the convalescent serum of COVID-19 patients. In addition, the immune serum exhibited a broad-spectrum immune response and was able to neutralize 10 different SARS-CoV-2 strains. No lung injury and no ADE effect was observed after virus challenge in rhesus monkeys, indicating the safety and high-level protection of the vaccine. Later, Sinovac Biotech published data from the phase 2 clinical trial of CoronaVac [59]. The vaccine was well tolerated, and there were no safety risks associated with vaccination. The adverse reactions were relatively mild, and no adverse reactions above grade 3 occurred. A phase 3 trial of CoronaVac is currently being carried out in Brazil, Indonesia and Turkey.

Using a special inactivation process, we developed an inactivated vaccine that uses a multi-epitope antigen with multiple exposed structural protein components of SARS-CoV-2 [60]. The vaccine induces neutralizing antibodiesin mice and rhesus monkeys witha dose-dependent relationship. Compared with the placebo and control vaccine, for which the RBD polypeptide was used as an antigen, the vaccine was able to completely inhibit viral replication in tissues and significantly reduced lung inflammation. Although neutralizing antibody titer is a widely accepted indicator of vaccine-induced effective antiviral immunity, our study showed that anti-S antibodies and anti-N antibodies may play a role similar to that of neutralizing antibodies in convalescent serum. The S and N proteins in viral seeds were exposed as antigens because of our inactivation process. When rhesus monkeys were challenged with SARS-CoV-2, a specific immune response was triggered by the vaccine, and neutralizing antibody titers increased with vaccine doses, as well as specific cytotoxic lymphocyte (CTL) responses to S, N and virus particles, andanti-N antibodies are especially closely related to the general antibody reaction. Anti-S and anti-N antibodies were produced in all animals. Intriguingly, we observed that even in the low-dose group, the vaccine induced protection, and the CTL response was similar to that in the high- and medium-dose groups, although the neutralizing antibody levels were low. In contrast, the immune response induced by the control RBD polypeptide vaccine in rhesus monkeys did not completely inhibit viral proliferation in some tissues, even with high neutralizing antibody titers, and pathological inflammation in the lung was slightly more serious than that in the high- and middle-dose groups. These results suggest that our inactivated vaccine provides systemic protection by elevating neutralizing antibody titers and increasing CTL responses associated with anti-N antibody levels. The major viral antigen N protein may activate the innate immunity of epithelial cells by interacting with intracellular pattern recognition receptors (PRRs), further promoting the specific antiviral immune response. While the S protein can induce neutralizing antibodies, the degree of interaction with PRRs is less than that with the N protein. Therefore, clinically protective immunity against SARS-CoV-2 infection should include not only specific neutralizing antibodies but also specific cellular immune responses to at least the S and N antigens. In a phase 1 clinical trial, we conducted extensive studies on the safety and immunogenicity of the vaccine [61]. The most common adverse reactions within 28 days after boostervaccination were mild pain and redness at the injection site or mild fatigue. There were no abnormal changes in 48 cytokines in immune serum samples, and seroconversion was associated with increased binding antibody against S protein, N protein and whole-virion, as well as the CTL reaction. In addition, the immune serum was diluted from 1:32 to 1:4096 and showed no ADEof human natural killer cells, macrophages, or dendritic cells. These preliminary results demonstrated the safety and immunogenicity of the vaccine. At present, a phase 1b/2b trial is being carried out in Sichuan, China.

## Other vaccine candidates

### Single nasal drip vaccine

Hassan et al. [62] developed a vaccine that uses the same ChAd vector as Oxford’s ChAdOx1 but is administered *via*a single nasal drip. A prefusion conformation of the S protein gene was inserted into the ChAd vector. When hACE2 mice were intramuscularly inoculated with the vaccine, the vaccine induced significant humoral and cellular immunity and prevented lung infection, inflammation and pathological damage after SARS-CoV-2 challenge but did not completely protect mice against SARS-CoV-2 infection. However, when the mice were inoculated by a single nasal drip, strong mucosal IgA and T cell responses were induced, and SARS-CoV-2 infection was completely prevented upon challenge. This study provides a simple and highly effective preventive vaccine, which is of great significance for the popularization of the vaccine in areas with poorly developed medical resources,as well as in infants and young children.

### Attenuated live vaccine

Chungnam National University published the first research on an attenuated SARS-CoV-2 vaccine [63]. A cold-adapted virus strain was constructed that did not cause weight loss or death in K18-ACE2 mice. Six days after infection, only trace amounts of the virus were detected in the lungs, and the pulmonary pathology was mild. At the same time, the strain induced high-titer neutralizing antibodies, a strong cellular immune response and an IgA mucosal antibody response in K18-ACE2 mice. A single intranasal inoculation protected the mice from SARS-CoV-2 infection. The study suggests that this cold-adapted strain might be used as a nasal spray for human vaccination, although the response in nonhuman primates and humans requires further study.

In March 2020, Duke-NUS Medical School of Singapore reported a SARS-CoV-2 strain with a 382-bp deletion in the ORF8 regionthat may reduce the virulence of the virus [64]. The Δ382 strain was more replicative in vitro than the wild-type strain [64], but there was no difference in viral load in patients, suggesting that the deletion did not impair its ability to replicate. The results of a clinical retrospective study showed that the Δ382 strain appeared to be a mildly pathogenic form, and patients in the Δ382 group did not develop severe hypoxia or dyspnea [65]. This strain was first detected in Singapore on January 29, 2020, and later in Taiwan Province, China. Subsequently, ORF7b/8 deletions of different lengths (ranging from 62 bp to 345 bp) were detected in other regions, such as Australia, Bangladesh and Spain. It is suggested that the development of an attenuated live vaccine could start at ORF8.

The University of Hong Kong has reported a cellular adaptation variant of SARS-CoV-2 called Ca-Del Mut. Ca-Del Mut was cold-adapted on the basis of Del-mut-1 [66], which carrying a deletion of 30 bp between S1 and S2. The results showed that the replication efficiency of the strain in cells was higher than that of the wild-type strain and that Ca-Del Mut replicated effectively in respiratory system tissues in hamster models. However, Ca-Del Mut did not cause overt disease, nor did it cause a cytokine storm in hamsters. Importantly, the strain elicited a strong neutralizing antibody response in hamsters, which developed complete immunity to the wild-type SARS-CoV-2 strain. The study supports Ca-Del Mut as a potential attenuated vaccine and provides a strategy for the development of an attenuated SARS-CoV-2 vaccine [67].

### Broad-spectrum coronavirus vaccine

The coronavirus S protein RBD is a key antigen, but its immunogenicity as a vaccine is limited and needs to be optimized. A broad-spectrum vaccine to prevent infection of a range of betacoronaviruses, including SARS-CoV-2, MERS-CoV and SARS-CoV, has been reported by Dai et al. [68]. They first designed a dimeric form of the MERS-CoV RBD that fully exposed the neutralizing antibody epitope of the RBD. Mice were immunized three times and challenged with MERS-CoV. The vaccine induced good protection, reducing the viral load in mice and alleviating pneumonia. Then, more stable RBD-sc-dimers (tandem repeat single chains) were designed, which are more immunogenic. The SARS-COV-2 vaccine designed by this method induced a highly effective antibody response in mice, and the neutralizing antibody titer was increased by 10–100-fold. The RBD-sc-dimer is easy to produce at an industry scale, which provides a guarantee for its further clinical use.

### Bacillus Calmette-Guérin (BCG) vaccine

The Bacillus Calmette-Guérin (BCG) vaccine is a live attenuated vaccine that has been successfully used against tuberculosis for approximately 100 years. Some studies have suggested that the vaccine appears to “train” the immune system to recognize and respond to a variety of infections, enhancing the innate immune response to subsequent infections [69–71]. In a clinical trial, subjects who were vaccinated with a live attenuated yellow fever vaccine four weeks after BCG vaccination exhibited reduced peakvalues of viremia on day 5, and peripheral blood mononuclear cells isolated from these subjects produced higher levels of proinflammatory factors in response to various stimuli [72]. A series of recent studiesshowed that countries vaccinated with BCG have a lower mortality rate from COVID-19 than countries without BCG vaccination [73–77]. During the first 30 days of the outbreak, BCG vaccination was associated with a significant decline in confirmed cases and deaths, and countries that do not have universal BCG vaccination are more vulnerable to the COVID-19 pandemic than those that do. Therefore, scientists are now testing whether BCG vaccination can prevent or attenuate the COVID-19 pandemic. VPM1002 is developed based on the traditional BCG vaccine and has been proved to be safer than BCG in many clinical trials. At present, a number of phase 3 clinical trials (NCT04439045, NCT04387409, NCT04435379) are underway to verify whether VPM1002 can reduce the risk and severity of SARS-CoV-2 infection in multiple risk groups, such as health care workers, elderly individuals and front-line workers. These trials are expected to be completed by 2021.

## Discussion

According to the World Health Organization (www.who.int), as of November 3, 2020, there are currently 47 candidate vaccines under clinical evaluation and 155 candidate vaccines under preclinical evaluation [78]. Despite the unprecedented progress achieved in a very short period of time with the cooperation of academic, government and industry sectors, there are still many unknowns and challenges related to vaccine research and development.

The first challenge is the lack of animal models. Acute infectious diseases are most commonly modeled in small animals, such as mice; however, SARS-CoV-2 does not naturally infect mice since the mouse ACE2 receptor is not sensitive to SARS-CoV-2 infection [12]. At present, researchers have used transgenic [79] or adenovirus vector [80] technologies to introduce hACE2 into mice to construct humanized mice [81] or have screened for a mouse-adapted strain of SARS-CoV-2 [82, 83]. In addition, researchers have constructed a hamster model to simulate severe COVID-19 [84]. However, these models are often questioned with respect to their physiological relevance [85]. Nonhuman primates mimic the physiology of humans infected with SARS-CoV-2 as closely as possible. Although researchers have developed some nonhuman primate infection models, such as rhesus monkeys with SARS-CoV-2 respiratory tract infection [86] and rhesus monkeys with SARS-CoV-2 infection via the conjunctival route [87], the information on infection provided by animal models is still far from sufficient.

Another issue that needs to be addressed is the lack of uniform criteria for evaluating vaccine efficacy. Phase 3 clinical trials that evaluate vaccine protection in normal vaccine development typically take at least one to several years; however, in the face of an emerging global pandemic, there is an urgent need to establish a standard for evaluating vaccine efficacy in the absence of phase 3 clinical trial data. The infectious disease vaccines currently on the market exert protection by inducing neutralizing antibodies, so most vaccine-developing institutions use neutralizing antibody titers as the most important outcome measure in published data. However, the use of different testing methods, units and statistical methods in different institutions makes it difficult to perform a truly parallel comparison of results, and even if the same method is used, the results will vary significantly among different virus strains used. Prior to the establishment of a standardized procedure, incorporating neutralizing antibody titers of convalescent serum as a reference in the trial design would facilitate a cross-sectional comparison of vaccine efficacy. In addition, the timing of testing, the type of antibody, the targeted epitopes and the neutralization titer are all crucial for the protective ability of antibodies [88], and the ability of antibodies to neutralize viruses in vitro is sometimes inconsistent with in vivo observations [89], so antibody production does not necessarily mean that the vaccine is protective [90]. Therefore, despite the urgent need for vaccines, long-term, rigorous and scientific phase 3 clinical trials are needed to truly assess the protective effects of vaccines.

The third challenge is that the study of SARS-CoV-2 pathogenesis is far from complete. Although studies have shown that the SARS-CoV-2 mutation strains such as D614G [91] can be neutralized by the serum of vaccinated animals and old strain virus infection-recovered patient antisera [35, 92, 93], we cannot ignore the possibility that viral evolution may lead to the ineffectiveness of a vaccine. In addition, case reports of reinfection with different strains have also indicated the requirement for broad-spectrum vaccine protection [88]. Besides, the S protein, the major antigen currently used in most vaccines, has an unstable prefusion conformation and stable fusion conformation. Some researchers have designed a stable prefusion conformation of S protein whichexhibited improved immunogenicity [94]. While structural analysis by cryo-electron microscopy and scanning electron microscopy (SEM) demonstrated that formaldehyde inactivation stabilized the S protein in the prefusion state [95], the impact of manufacturing processes on the stability and immunogenicity of vaccines is likely to pose challenges for future vaccine mass production and long-term preservation.

Finally, SARS-CoV-2 is similar to SARS-CoV in all respects, and there is some evidence of ADE with SARS-CoV in animal studies [96–98] and in individual clinical cases [97, 99]. The existence of the ADE effect of SARS-CoV-2 has been one of the major concerns in candidate vaccine development. Although antibodies that can promote the entry of pseudoviruses into cell lines expressing Fc receptors in vitro have been demonstrated to exist in the serum of convalescent patients [100], to date, in vivo studies have not supported the existence of ADE effects [101–105]. According to our research (unpublished), inactivated vaccine serum does not induce ADE-mediated virus proliferation in macrophages, dendritic cells (DCs) or natural killer (NK) cells in vitro. In summary, there is simply not enough evidence to know whether there is ADE with SARS-CoV-2, so it remains one of the issues that warrants close attention in the continued development of vaccines.

## Conclusions

With the phase 3 clinical trials of several vaccines, including Pfizer, Moderna, AstraZeneca, etc. are expected to yield results soon, the production capacity and equitable distribution of these vaccines are the top priorities right now. In the meantime, facing the challenges of suitable animal models, uniform criteria for evaluating vaccine efficacy, deeper understanding of SARS-CoV-2 pathogenesis as well as further exploration for the evidence of ADE are becoming more and more important. Addressing these issues would ultimately improve COVID-19 vaccinesin the long run, which could be the fastest-applied vaccines in the history of human-fighting-pandemic.

## Data Availability

All data generated or analysed during this study are included in this published article.

## Abbreviations

ACE2: Angiotensin converting enzyme 2
ADE: Antibody-dependent enhancement
COVID-19: Coronavirus disease 2019
CTL: Cytotoxic T lymphocyte
DCs: Dendritic cells
E protein: Envelope protein
FP: Fusion peptide
GMT: Geometric mean titers
HR: Heptad repeats
IFN-γ: Interferon gamma
IMBCAMS: Institute of Medical Biology, Chinese Academy of Medicine Sciences
LNP: Lipid nanoparticle
M protein: Membrane protein
MERS-CoV: Middle East respiratory syndrome-related coronavirus
N protein: Nucleocapsid protein
NSP: Nonstructural proteins
NTD: N-terminal domain
ORF8: Open reading frame 8
PRRs: Pattern recognition receptors
RBD: Receptor binding domain
S protein: Spike protein
SAE: Serious vaccine-related adverse events
SARS-CoV: Severe acute respiratory syndrome coronavirus
SARS-CoV-2: Severe acute respiratory syndrome coronavirus 2
SEM: Scanning electron microscopy
SP: Signal peptide
WIBP: Wuhan Institute of Biological Products
